# Recurrence of Sigmoid Volvulus Associated With Constipation: A Retrospective Cohort Study

**DOI:** 10.7759/cureus.68972

**Published:** 2024-09-09

**Authors:** Chihiro Uda, Kei Tsumura, Chiaki Sano, Ryuichi Ohta

**Affiliations:** 1 Family Medicine, Fuchu Hospital, Osaka, JPN; 2 Community Medicine Management, Shimane University Faculty of Medicine, Izumo, JPN; 3 Community Care, Unnan City Hospital, Unnan, JPN

**Keywords:** colonic diseases, constipation, logistic models, recurrence, risk factors, sigmoid volvulus

## Abstract

Introduction

Sigmoid volvulus is a gastrointestinal condition characterized by twisting the sigmoid colon, leading to obstruction and potentially severe complications. It is associated with factors such as advanced age, constipation, and the use of certain medications. Despite treatment, recurrence is common and significantly impacts patients' quality of life. This study aimed to identify factors influencing the recurrence of sigmoid volvulus to develop effective preventive strategies.

Methods

A retrospective cohort study was conducted at Fuchu Hospital, Osaka, including 44 patients diagnosed with sigmoid volvulus between May 2013 and May 2023. Data on variables such as age, gender, constipation, cardiac and neuropsychiatric diseases, hypertension, diabetes mellitus, sigmoid colon overgrowth, and BMI were collected from electronic medical records. Recurrence was defined as two or more diagnoses of sigmoid volvulus during the study period. Logistic regression analysis was used to identify significant predictors of recurrence.

Results

Of the 44 patients, 20 experienced recurrences. Single regression analysis identified constipation, neuropsychiatric disorders, and sigmoid colon overgrowth as significant factors. Logistic regression analysis confirmed constipation as an important predictor of recurrence (OR: 8.84, 95% CI: 2.05-38.1, p=0.0034). The area under the receiver operating characteristic (ROC) curve for the model was 0.804 (95% CI: 0.67-0.938), indicating good predictive accuracy.

Conclusion

Constipation is a significant risk factor for the recurrence of sigmoid volvulus, likely due to chronic fecal overload leading to elongation and dilation of the sigmoid colon. Effective management of constipation is crucial in preventing recurrence. Future prospective studies with larger sample sizes are needed to validate these findings and explore additional preventive measures.

## Introduction

Sigmoid volvulus is a remarkably complex mechanism of gastrointestinal disease, accounting for 60% to 80% of all colonic volvulus cases globally, and it affects approximately 2.5 to 7.0 people per 100,000 annually [1]. The disease is known to be associated with a variety of factors, including chronic constipation, advanced age, and the use of certain medications such as antipsychotics [2]. Constipation may lead to an abnormal increase in intra-abdominal pressure and torsion of the colon, particularly in patients with elongated colons or mesentery [3]. Numerous studies have shown that the incidence of sigmoid volvulus tends to be higher in patients aged 70 years or older, accounting for up to 70% of cases worldwide, with a significant prevalence observed in elderly patients and those on antipsychotic medications [3,4]. Thus, sigmoid volvulus is a multifactorial disease, and understanding the process leading to its onset is crucial for developing effective preventive measures.

Colon volvulus can recur even once treated, and recurrence can seriously affect a patient's quality of life (QOL). Recurrence forces the patient to undergo another surgery or prolonged hospitalization, increasing physical, emotional, and financial burdens [5]. Therefore, preventing recurrence is critical in protecting patients' health and quality of life [6]. However, research to date is poorly understood regarding the mechanisms of recurrence and the risk factors that promote it, and this knowledge gap impedes the development of recurrence prevention strategies.

This study aimed to identify factors influencing the recurrence of sigmoid volvulus. A retrospective cohort study of first-episode patients was conducted to identify variables that may increase the risk of recurrence through logistic regression analysis. Findings from this study are expected to contribute to developing new interventions and improved treatment guidelines to prevent sigmoid colon axis torsion recurrence.

## Materials and methods

Fifty-one patients diagnosed with sigmoid volvulus at our hospital were included between May 2013 and May 2023. The primary endpoint was recurrence of sigmoid volvulus. Independent variables were selected based on the selection literature: elderly over 75 years, gender, constipation, cardiac disease, neuropsychiatric disease, hypertension, diabetes mellitus, sigmoid colon overgrowth, and BMI < 18.5. Recurrence was defined as having two or more diagnoses of sigmoid volvulus during the study period. Constipation was defined as a diagnosis of constipation or chronic constipation by a previous physician or our clinic before the diagnosis of sigmoid volvulus.

Setting

Fuchu Hospital in Osaka is a prime example of a medical education setting. Situated in a city known for dynamism, the hospital has state-of-the-art facilities, including high-tech operating rooms and interactive learning centers. Its affiliation with local medical schools fosters a robust educational environment, facilitating knowledge exchange between experienced clinicians and medical students.

The hospital's comprehensive clinical services, covering various specialties, underscore its educational framework. This approach emphasizes a multidisciplinary methodology and patient-centric care. Cutting-edge technology, such as robotic-assisted surgery and telemedicine, significantly enhances treatment and learning outcomes.

As a central secondary hospital in the Senshu secondary medical care area, Fuchu Hospital is responsible for emergency medical care in the northern part of Senshu [7,8].

The target diseases are general internal medicine and trauma, except multiple trauma. In 2020, 5,126 emergency cases were transported, and 2,063 cases were hospitalized, for a hospitalization rate of 40.2%. The Izumi City Fire Department transported 2,925 cases and the Izumiotsu City Fire Department 1,408 cases, followed by Kishiwada City, Tadaoka Town, Takaishi City, and Sakai City.

Fuchu Hospital also prioritizes cultural competence and international collaboration, creating a welcoming environment for diverse communities. The hospital extends its educational impact beyond its walls through extensive community outreach programs, promoting health awareness in the broader Osaka region. Thus, the institution is not merely a healthcare facility but also a hub for nurturing future medical professionals in an environment that mirrors the intricacies and compassion of modern medicine.

Participants

This study's target population consisted of patients who visited Fuchu Hospital between May 2013 and May 2023 and had been previously diagnosed and treated for sigmoid volvulus.

Patients with a second diagnosis of sigmoid volvulus at Fuchu Hospital during the above period and those with a first diagnosis of sigmoid volvulus at a hospital other than Fuchu Hospital. To investigate recurrence, patients for whom complete follow-up was unavailable, as well as patients who had undergone surgical treatment for sigmoid volvulus, were also included. Exclusion criteria were patients with missing information in the electronic medical record.

Measurements

Data were collected retrospectively from hospital electronic medical records. The nine independent variables were as follows: age older than 75, gender, constipation, cardiac disease, neuropsychiatric disease, hypertension, diabetes mellitus, sigmoid colon overgrowth, and BMI less than 18.5 [9].

Methods of analysis

For continuous variables, the normality of the data was checked before statistical tests. Logistic regression analysis was performed to examine the correlation between the recurrence of sigmoid volvulus and influencing factors. The multivariate logistic model considered only variables with a significant trend in the single-split analysis. All statistical analyses were performed using Easy R with a significance level of p<0.05 [10].

Ethical considerations

The presence or absence of recurrence in the medical record was detected for 10-year sigmoid volvulus in all 51 cases. The data were stored so that the medical record numbers and patients' names could not be identified. This study was approved by the ethics committee of Fuchu Hospital (Ethics Committee approval number: 2024012).

## Results

Patient background

The study compared various factors between the recurrence group (n=20) and the non-recurrence group (n=24) to identify potential predictors of recurrence. The analysis revealed significant differences between the two groups regarding constipation, with 70.0% of the recurrence group experiencing constipation compared to only 20.8% in the non-recurrence group (p=0.002). This suggests that constipation may be a strong predictor of recurrence. Other factors, including neuropsychiatric disorders, sigmoid colon overgrowth, gender, age, BMI, hypertension, heart disease, and diabetes, did not show statistically significant differences between the two groups. Specifically, neuropsychiatric disorders were present in 35.0% of the recurrence group and 16.7% of the non-recurrence group (p=0.185). Sigmoid colon overgrowth was observed in 25.0% of the recurrence group compared to 4.2% of the non-recurrence group (p=0.077). Regarding gender distribution, 45.0% of the recurrence group were male and 55.0% were female while the non-recurrence group comprised 62.5% males and 37.5% females (p=0.363). Age distribution was similar between the two groups, with 65.0% of the recurrence group being 75 years or older compared to 66.7% in the non-recurrence group (p=1.000). BMI under 18.5 kg/m^2^ was noted in 40.0% of the recurrence group and 29.2% of the non-recurrence group (p=0.532). Hypertension was present in 55.0% of the recurrence group and 45.8% of the non-recurrence group (p=0.763). Heart disease was reported in 30.0% of the recurrence group and 29.2% of the non-recurrence group (p=1.000). Lastly, diabetes was observed in 15.0% of the recurrence group and 25.0% of the non-recurrence group (p=0.477) (Table 1).

**Table 1:** Demographics of the participants BMI: body mass index, Recurrence: having two or more diagnoses of sigmoid volvulus during the study period. Student’s t-test was used to analyze parametric data, whereas the Mann–Whitney U test was used to analyze nonparametric data.

Factor	Recurrence group	Non-recurrence group	p-value
N (%)	20	24	
Male	9 (45.0)	15 (62.5)	0.363
Female	11(55.0)	9(37.5)	
75 years old and over	13 (65.0)	16 (66.7)	1.000
Under 75 years old	7(35.0)	8(33.3)	
BMI (kg/m^2^) less than 18.5	8 (40.0)	7 (29.2)	0.532
BMI (kg/m^2^) over 18.5	12(60.0)	17(70.8)	
Constipation	14（70.0）	5（20.8）	0.002
Neuropsychiatric disorders	7（35.0）	4（16.7）	0.185
Sigmoid colon overgrowth	5 (25.0)	1 (4.2)	0.077
Hypertension	11 (55.0)	11 (45.8)	0.763
Heart disease	6 (30.0)	7 (29.2)	1.000
Diabetes	3 (15.0)	6 (25.0)	0.477

Logistics regression analysis

A multivariate logistic regression analysis investigated factors associated with recurrent sigmoid colon axis torsion. The analysis focused on three variables-constipation, neuropsychiatric disorders, and sigmoid colon overgrowth, which showed a tendency toward significance in the univariate analysis. Constipation was significantly associated with recurrent sigmoid volvulus, with a P value of 0.0034 (Table 2).

**Table 2:** Result of the logistic regression analysis

	Odds ratio	95% confidence interval	P-value
Constipation	8.84	2.05-38.1	0.0034
Neuropsychiatric disorders	2.54	0.45-14.2	0.288
Sigmoid colon overgrowth	4.32	0.37-50.0	0.241

**Figure 1:**
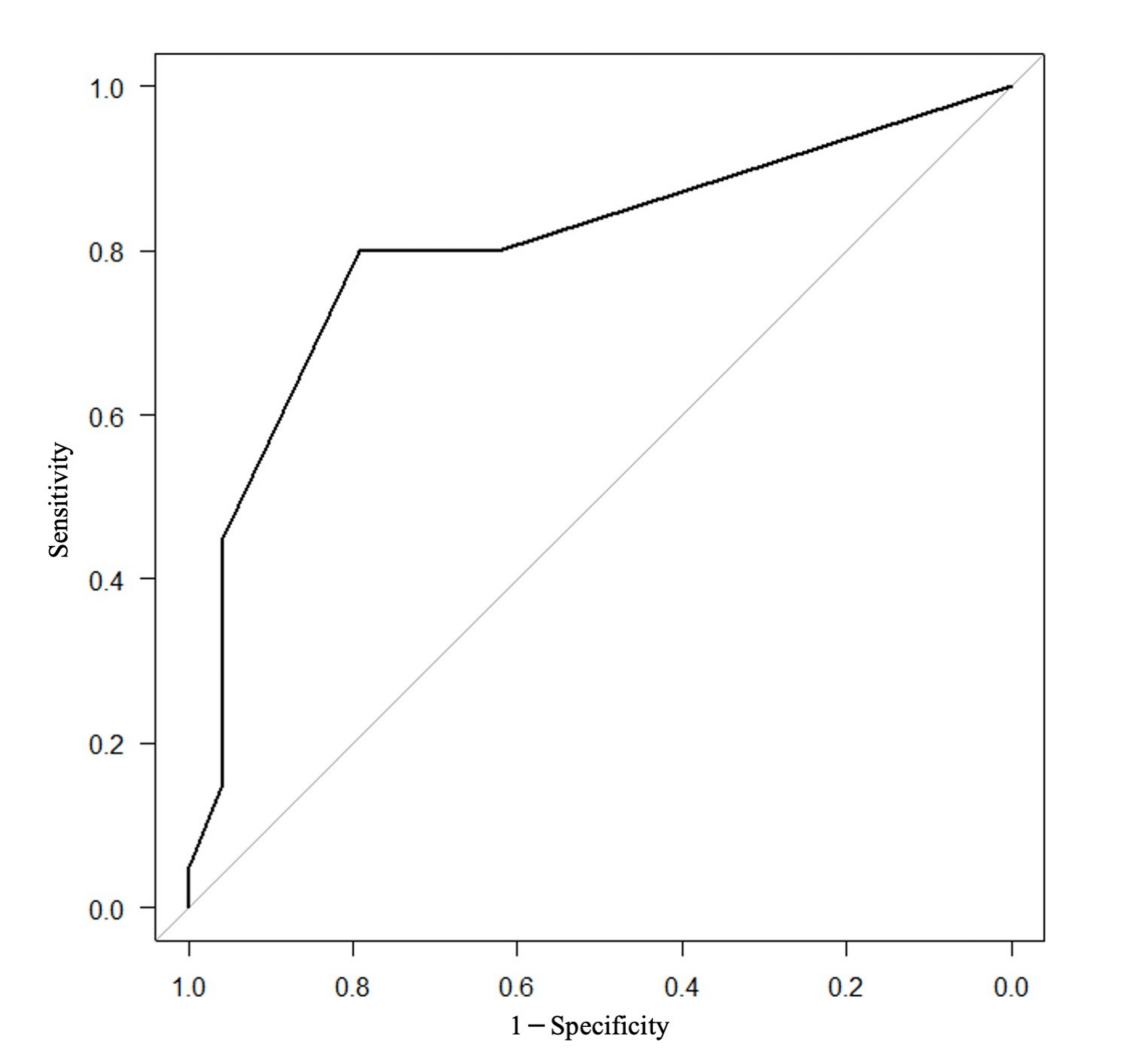
Receiver operating characteristic (ROC) curve Illustrating the performance of the logistic regression model in predicting the recurrent sigmoid colon axis torsion The gray line shows the case where the relationship between the independent variable and the outcome is completely coincidental. Also, the black line shows the results of the current study.

The area under the ROC curve was 0.804 (95% confidence interval: 0.67-0.938) (Figure 1).

## Discussion

This study showed a significant trend for sigmoid colon overload and constipation. Based on this, logistic regression analysis indicated that constipation was a significant factor. These results suggest that constipation predisposes to sigmoid colon overgrowth and is a recurrent factor in sigmoid volvulus. The interaction of intestinal anatomy and chronic fecal overload can explain the impact of constipation on the development and recurrence of sigmoid volvulus. Therefore, managing constipation is essential for preventing sigmoid volvulus and its recurrence.

Sigmoid volvulus occurs when the air-filled loop of the sigmoid colon twists into the mesentery [11]. The anatomy of this condition is characterized by the loose hoofing of the sigmoid colon and the proximity of its attachment points to the retroperitoneum, which predisposes it to torsion [12]. This freedom contributes to the increased risk of torsion. The sigmoid colon is not fixed to the retroperitoneum, making it a particularly vulnerable site for axial torsion. It has been reported that approximately 90% of all colonic axis torsions occur in the sigmoid colon [13]. Regarding the degree of torsion, a torsion greater than 180 degrees obstructs the intestinal lumen, and a torsion greater than 360 degrees leads to obstruction of vascular return [14]. This increases the risk of intestinal ischemia and necrosis. In addition, there is a variant of sigmoid volvulus called sigmoid ileum [15]. In this case, it occurs when the ileum wraps around the sigmoid colon, usually in a clockwise direction. The coiling of the ileum can cause further bowel obstruction and vascular compromise [16]. Thus, sigmoid volvulus is a complex condition that, depending on its anatomical features and degree of torsion, can obstruct the intestinal lumen and impair vascular return, requiring early diagnosis and appropriate treatment.

Sigmoid colon axis tortuosity is predisposed to occur due to specific anatomic features. These include a narrow mesenteric attachment site and a long, redundant sigmoid colon [9]. The following mechanisms may account for the influence of constipation on this condition. Concerning the relationship between anatomic features and constipation, the narrow mesenteric attachment of the sigmoid colon allows the sigmoid colon to move freely and is prone to torsion [17]. In addition to this, if the sigmoid colon is redundant, its length further increases the risk of torsion [18]. A redundant sigmoid colon also contributes to this; the long and redundant sigmoid colon forms a structure prone to torsion. This makes torsion more likely when fecal overload is added due to constipation. A possible mechanism by which constipation causes sigmoid colon axis torsion is elongation and dilation due to fecal overload [19]. Chronic constipation causes fecal accumulation in the intestine, which overloads the sigmoid colon. This causes elongation and dilation of the sigmoid colon. This elongation and dilation increase the risk of torsion. Furthermore, concerning the risk of recurrence, fecal overload due to constipation increases the risk of recurrence in patients who have once experienced sigmoid colon axis torsion [20]. Continued fecal overload increases the likelihood of another torsion.

This study has several limitations that need to be addressed. First, the retrospective design may introduce selection bias, as only patients diagnosed and treated at Fuchu Hospital were included. This limits the generalizability of the findings to other settings or populations. Second, the sample size of 44 patients is relatively small, potentially affecting the power of the study and the ability to detect significant associations. Third, data on potentially relevant variables, such as dietary habits and physical activity levels, were unavailable, which might have provided further insights into the recurrence of sigmoid volvulus. Additionally, the reliance on electronic medical records may lead to incomplete or inaccurate data, especially regarding the diagnosis and management of constipation and other comorbid conditions. Lastly, the follow-up period varied among patients, which could influence the assessment of recurrence rates.

## Conclusions

This study identified constipation as a significant risk factor for the recurrence of sigmoid volvulus, highlighting the importance of managing constipation in patients with this condition. The findings suggest that chronic fecal overload and the resulting elongation and dilation of the sigmoid colon increase the risk of torsion and recurrence. These results underscore the need for proactive measures to address constipation, such as dietary modifications, pharmacological interventions, and regular follow-up, to prevent recurrence and improve patient outcomes. Future studies with larger sample sizes and prospective designs are warranted to validate these findings and explore additional risk factors and preventive strategies. By enhancing our understanding of the mechanisms underlying sigmoid volvulus recurrence, we can develop more effective interventions and treatment guidelines to reduce the burden of this condition on patients and healthcare systems.
